# Gastrointestinal Cryptococcosis Associated with Intestinal Lymphangiectasia

**DOI:** 10.1155/2020/7870154

**Published:** 2020-04-20

**Authors:** Fernando Naranjo-Saltos, Alejandro Hallo, Carlos Hallo, Andres Mayancela, Alejandra Rojas

**Affiliations:** ^1^Internal Medicine Department, Hospital de Especialidades Eugenio Espejo, Quito, Ecuador; ^2^Internal Medicine Department, NYU-Winthrop Hospital, Mineola, NY, USA

## Abstract

Intestinal lymphangiectasia is a pathological dilation of enteric lymphatic vessels resulting in lymph leakage to the intestinal lumen. This chronic lymph leakage leads to a state of immunosuppression secondary to the loss of humoral and cellular components of the immune system and represents a potential risk factor for opportunistic infections. We report a case of protein-losing enteropathy in a seemingly immunocompetent patient. An intestinal histopathological study revealed the unusual association of lymphangiectasia and intestinal cryptococcosis. Although cryptococcal infection is common in immunocompromised patients, intestinal involvement is rarely reported. We found no reports on the association of intestinal cryptococcosis in patients with lymphangiectasia. This case report is the first to describe intestinal cryptococcosis associated with intestinal lymphangiectasia.

## 1. Case

A 37-year-old man presents to the Emergency Department with abdominal pain exacerbation of 3 hours of evolution associated diarrhea with melena. The patient had a documented medical history of intermittent abdominal pain and diarrhea over the 6 months prior to the current admission. Upon admission, the patient presented febrile and in anasarca, with major edema in lower limbs. Initial blood work showed moderate microcytic anemia (Hb 10.3 g/dl, Hct. 23.7%, and MCV 75 fL), severe hypoalbuminemia (1.7 g/dL), inversion of albumin/globulin ratio, and CBC did not show signs of infection.

The patient was started on protein replenishment with human albumin which resolved partially the edema.

An exhaustive workout was performed to identify the underlying cause of the edema and low protein levels. Liver disease was ruled out due to normal liver profile (total bilirubin 0.21 mg/dL, indirect bilirubin 0.11 mg/dL, direct bilirubin 0.10 mg/dL, AST 9 IU/L, and ALT 8 IU/L) and the negative liver ultrasound. Nephrotic syndrome was also ruled out as a possible diagnosis.

Due to the persistence of abdominal pain, structural damage or tumor processes were investigated; however, abdominal CT only showed edema in the small intestine wall suggesting chronic enteropathy.

Although the patient was considered initially immunocompetent, there was a significant drop in complement C3 0.5 g/L (negative <0.8 g/L) and C4 0.03 g/L (negative <0.10 g/L). Further laboratory testing showed hypogammaglobulinemia (22 mg/dL IgA (normal 22–149 mg/dL), 150 mg/dL IgG (normal 615–1530 mg/dL), 20 mg/dL IgM (normal 31–272 mg/dL)), and 20 mL/24 hrs *α*-1 antitrypsin clearance.

An upper digestive endoscopy was performed showing a whitish lace pattern and erythematous walls in the second portion of the duodenum. In addition, a colonoscopy showed hemorrhoidal packages, multiple ulcerated lesions in sigma, and elevated, pseudopolypoid, infiltrative lesions in cecum and ileocecal valve.

Since our patient lived in a tuberculosis endemic area and that the lesions simulated intestinal tuberculosis, empirical antituberculosis therapy (isoniazid 75 mg, rifampicin 150 mg, ethambutol 275 mg, and pyrazinamide 400 mg) was started until biopsy results came back.

**Figure 1:**
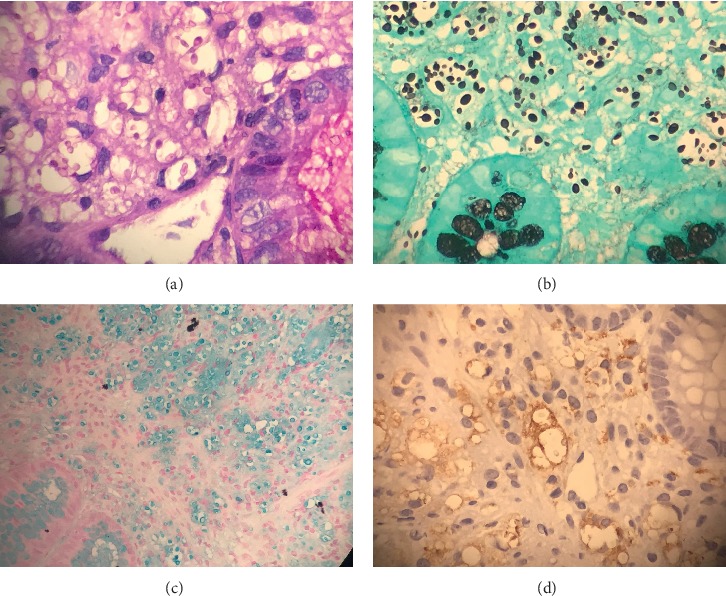
Macrophages loaded with cryptococcal spores (arrowhead): (a) PAS staining, (b) Grocott staining, (c) Alcian blue staining, and (d) immunohistochemistry CD68.

**Figure 2:**
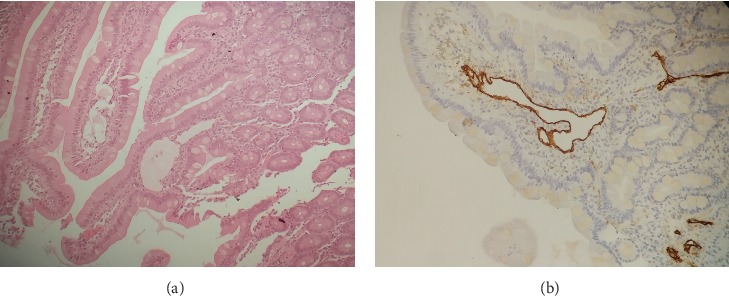
Histopathological biopsy study obtained from the lower gastrointestinal tract. (a) Lymphangiectasia in lamina propria. (b) Lymphangiectasia highlighted with D240 immunostaining (arrowhead).

Multiple cryptococci were found in samples taken from the cecum, sigma, and ileocecal valve using the techniques of immunohistochemistry CD68, Grocott stain, PAS stain, and Alcian blue stain (Figure 1). In addition, the presence of intestinal lymphangiectasia was confirmed with D240 staining (Figure 2).

After receiving the pathology report, antituberculosis therapy was suspended and a regimen consisting of fluconazole 800 mg/day IV for 2 weeks, and octreotide 1 ml subcutaneously every 8 hours was initiated. Due to the severity of the presentation octreotide was started along with dietary treatment. Octreotide and oral fluconazole 600 mg were maintained for 90 days after discharge with favorable symptomatic evolution, additionally, following laboratory controls shown that albumin levels went back to normal.

## 2. Discussion

Cryptococcosis is one of the most frequent opportunistic fungal diseases. Its incidence in Latin America has been increasing, reaching approximately 5300 cases in 2017. However, intestinal dissemination is rarely reported [1–3].

An exhaustive literature review in the Medline databases was performed using the terms “gastrointestinal cryptococcosis” and “intestinal lymphangiectasia.” We identified a total of 10 case reports of intestinal cryptococcosis in the context of immunosuppressing conditions which are summarized in Table 1; however, we were unable to find reports similar to our case. The lack of literature on the coalescence of these conditions suggests that this work is the first description of intestinal cryptococcosis associated with intestinal lymphangiectasia.

The countries with the highest incidence of common presentations of Cryptococcosis are Brazil, Colombia, and Venezuela. The infection occurs through contact with soil or eucalyptus trees contaminated with birds' feces. Most of these cases occur in young male patients diagnosed with HIV/AIDS, and in much lesser proportion in immunocompetent patients [2, 3].

The infection involves the central nervous system (CNS) in almost 70% of cases, being the most common cause of meningitis in HIV/AIDS patients. Lung involvement is the second most common presentation and presents as infiltrations or granulomatous reactions [2, 3, 9].

The increasing prevalence of patients diagnosed with AIDS has been proportional to the increase in opportunistic infections such as Cryptococcosis. The use of antiretroviral therapy (ART) has modified its presentation, resulting in atypical locations. Intestinal cryptococcosis is believed to be through direct inoculation rather than hematogenous dissemination since blood cultures were always negative [2, 9].

The apparent immunocompetence of our patient and his unspecific symptomatology made his diagnosis a real challenge, misguiding the diagnostic suspicion towards more common gastrointestinal tract infections such as tuberculosis. Similar situations happened in a large number of reported cases of intestinal cryptococcosis, in which diagnosis occurred incidentally.

Out of 11 case reports of gastrointestinal cryptococcosis, only Sciaudone G presented an immunocompetent patient while the rest reported immunologically compromised patients. The correlation between immunocompromised and intestinal cryptococcosis is well documented. Patients with advanced stages of HIV (CD4 < 200), with malignant processes (solid or hematological tumors), treatment with immunosuppressants [4, 6], organ-transplanted [11], or cirrhotic [9, 12] are considered as high-risk subjects.

Intestinal lymphangiectasia can produce a state of secondary immunodeficiency. The pathological dilation of lymphatic vessels in the intestinal wall, especially in the submucosa, causes chronic loss of lymph containing immunoglobulins and lymphocytes (predominantly naïve CD4 and CD8 T cells) and severe hypoalbuminemia. This state of secondary immunodeficiency is of difficult diagnosis and poses a high risk for opportunistic fungal infections such as cryptococcosis, as in our patient [13, 14].

Our patient's age was accord with the average age of intestinal cryptococcosis presentation reported in the literature (37 years, ranging from 26 to 73 years) [3].

Abdominal pain was the most common initial symptom in patients infected with Cryptococcus. The onset of pain in reported cases ranged from 2 days to 6 months; however, the majority exceeded 3 weeks. In addition, 66% of patients had fever at some point in the course of the illness associated with vomiting and nausea sporadically. Despite being an invasive intestinal pathology, only half of the patients had diarrhea, and only 25% had bloody diarrhea or melena. Any gastrointestinal organ can be affected.

Through endoscopy, suggestive lesions, as ulcers or abscesses, and signs of inflammation can be evidenced. However, ulcers can be similar to those produced in malignancy, other infections (Cytomegalovirus, *Toxoplasma gondii*, *Leishmania donovani*, etc.), or Crohn's disease [3, 7, 11, 15].

Although management varies depending on the localization of infection, fluconazole 400 to 800 mg once daily for 8 weeks followed by medium doses for 12 months can be used empirically. Intravenous amphotericin B is considered as the first line of treatment for disseminated Cryptococcus; however, its use in gastrointestinal cryptococcosis is not well studied (F. Cicora) (Rupashree Sundar).

The cryptococcal infection had high mortality rates in immunocompromised patients; however, it is important to consider that in the majority of reports the patient's general condition was already severely compromised due to the advanced state of the underlying disease prior to infection.

## Figures and Tables

**Table 1:** Case reports of intestinal cryptococcosis in the context of immunosuppressing conditions.

	Immune status	Age	Presentation	Endoscopy	Place of infection
Case 1	Immunocompetent	37	Abdominal pain (6 months), melena, fever	Ulcerated and elevated lesions	Sigma, blind and ileocecal valve
Chavapradit and Angkasekwinai [4]	Immunosuppressive therapy	64	Abdominal pain	Inflammation of the mucosa, whitish exudates	Blind, ascending colon
Eyer-Silva et al. [5]	HIV infection (CD4 10/mm^3^)	34	Abdominal pain (2 months), nausea and vomiting	High lesions flushed with central ulcer	Stomach
Osawa and Singh [6]	Immunosuppressive therapy	53	Intermittent abdominal pain, fever, and diarrhea	Linear ulcer	Ileus terminal
Sundar et al. [7]	HIV infection (ART not started)	48	Uncontrollable vomiting (3 days)	Macroscopic erosion	Stomach
Liu [8]	AIDS	54	Fever, diarrhea, and fever (8 days)	Irregular ulcers, violet pigmented lesions	Stomach, duodenal bulb and second portion of the duodenum
Musubire et al. [9]	HIV infection (CD4 5 cells/mL)	37	Abdominal pain fever	Lymphadenopathy	Ileus
Girardin et al. [10]	HIV infection (3 cells/mL)	26	Epigastric pain (3 weeks), bilious vomiting, fever	Patched lesions, with whitish villi	Duodenum
Cicora et al. [11]	Immunosuppressive therapy	59	Diarrhea	Unique ulcer	Large intestine
Sciaudone et al. [3]	Immunocompetent	26	Abdominal pain, fever, diarrhea, and melena	Hyperemic mucosa, ulcer	Sigmoid colon
Hokari et al. [12]	Primary biliary cirrhosis	58	Fever, diarrhea	Pseudopolyposis	Small and large intestine

Association	Image	Complications	Management		

Lymphangiectasia	CT: small bowel edema	Without complications	Fluconazole 800 mg IV		
Crohn's disease	Thickening and edema of cecum, ileocecal valve, and terminal ileum	Dissemination	Amphotericin B 0.7 mg/kg daily for 6 weeks. Fluconazole 200 mg/day for one year		
Meningoencephalitis	Not reported	Not reported	Amphotericin B followed by fluconazole		
Crohn's disease	No significant changes	Dissemination	Amphotericin B followed by fluconazole 40 mg day (19 days)		
Herpes simplex type I	Not reported	Not reported	Amphotericin B		
Sepsis	Ulcer at the level of the antrum with central reddish ulceration	Multiorgan failure	Amphotericin B, fluconazole, pantoprazole IV		
Not reported	Ultrasound: thickening of the ileum wall. Rx abdominal: signs of perforation	Not reported	Not reported		
Transplanted kidney	Not reported	Not reported	Amphotericin B (8 days), fluconazole 800 mg daily (3 months)		
Not reported	CT: hypertrophic right lobe in liver, thickening of the wall of the cecum, and transverse colon	Not reported	Fluconazole 400 mg daily (1 week); 200 mg (5 weeks)		
Liver dysfunction, pneumonia	CT: intestinal distention, fluid accumulation	Multiorgan failure	Antibiotics		
